# Association Between Physical Activity Levels and the Prevalence of Self-Reported Diabetes Among Adults in the United States: A Secondary Analysis of National Health and Nutrition Examination Survey (NHANES) 2005–2006

**DOI:** 10.7759/cureus.115553

**Published:** 2026-08-31

**Authors:** FNU Shweta, FNU Partab, Sameet Kumar, FNU Kashish, Anish Kumar, Prinka Kumari, Luksh Virwani, Nimarta Bheesham, Sohan Virwani

**Affiliations:** 1 Epidemiology and Biostatistics, Drexel University, Philadelphia, USA; 2 Internal Medicine, Chandka Medical College, Larkana, PAK; 3 Nephrology, University of Florida, Gainesville, USA; 4 Internal Medicine, Jinnah Sindh Medical University, Karachi, PAK; 5 Medical School, Ziauddin University, Karachi, PAK; 6 Pediatrics, Aga Khan University Hospital, Karachi, PAK; 7 Medical School, Jinnah Medical and Dental College, Karachi, PAK; 8 Internal Medicine, University of Illinois College of Medicine Peoria, Peoria, USA; 9 Medical School, Ghulam Muhammad Mahar Medical College, Sukkur, PAK

**Keywords:** body mass index, cross sectional studies, diabetes mellitus, national health and nutrition examination survey (nhanes), physical activity, self-reported diabetes

## Abstract

Introduction: Physical activity (PA) is an important modifiable lifestyle factor associated with metabolic health and diabetes. This study examined the association between different levels of self-reported PA and prevalent self-reported diabetes among adults in the National Health and Nutrition Examination Survey (NHANES) 2005-2006 sample.

Methods: This cross-sectional secondary analysis included 8,174 adults aged 18 years or older from NHANES 2005-2006. PA was categorized as low (<150 minutes/week), moderate (150-300 minutes/week), or high (>300 minutes/week). Diabetes status was based on self-reported physician diagnosis. Logistic regression models were used to examine associations between PA level and prevalent self-reported diabetes with sequential adjustment for demographic characteristics, socioeconomic factors, and body mass index (BMI).

Results: Overall, 650 participants (8.0%) had self-reported diabetes. Diabetes prevalence was 11.8% in the low PA group, 5.9% in the moderate group, and 5.7% in the high group. In the unadjusted model, moderate PA was associated with lower odds of diabetes (OR 0.47, 95% CI 0.36-0.61), as was high PA (OR 0.45, 95% CI 0.38-0.54). After adjustment for demographic and socioeconomic characteristics and BMI, the associations were attenuated but remained statistically significant for moderate activity (OR 0.75, 95% CI 0.57-0.99) and high activity (OR 0.81, 95% CI 0.66-0.99).

Conclusion: Moderate and high levels of self-reported PA were associated with lower odds of prevalent self-reported diabetes compared with low PA in the analyzed NHANES sample. Because of the cross-sectional design, temporality and causality cannot be established. Prospective studies using appropriate complex-survey methods are needed to further evaluate this association.

## Introduction

Diabetes is a major global public health concern. An estimated 537 million adults are living with diabetes worldwide, and this number is projected to increase substantially by 2030 [1,2]. In the United States, more than 38 million adults are affected by diabetes, while approximately 98 million adults have prediabetes [3]. Diabetes is associated with substantial morbidity, mortality, and economic burden, accounting for approximately $413 billion in annual direct and indirect healthcare costs in the United States [4].

Physical activity (PA) is an important modifiable lifestyle factor associated with diabetes risk and metabolic health. Previous studies have shown that individuals who engage in moderate to vigorous PA have lower rates of type 2 diabetes than those who are physically inactive [5,6]. Both aerobic and resistance exercise have also been associated with improvements in insulin sensitivity, glucose utilization, skeletal muscle function, and glycemic control [7,8]. Resistance training may increase skeletal muscle glucose uptake, while endurance exercise can improve oxidative metabolism and insulin sensitivity [9,10]. These findings support current recommendations encouraging regular aerobic and resistance activity as part of diabetes prevention and management strategies. The Diabetes Prevention Program further demonstrated that an intensive lifestyle intervention incorporating PA, dietary modification, and weight management was associated with a greater reduction in diabetes incidence than metformin among individuals at high risk for diabetes [11].

Although a substantial body of literature has demonstrated an association between PA and diabetes, it is important to examine how different levels of PA are associated with prevalent diabetes in population-based datasets after accounting for demographic and socioeconomic characteristics. In particular, comparison of low, moderate, and high activity categories may help clarify whether individuals reporting greater levels of activity also have lower odds of prevalent diabetes. Therefore, the present study was conducted as a cross-sectional secondary analysis of the National Health and Nutrition Examination Survey (NHANES) 2005-2006 data to examine the association between PA levels and prevalent diabetes among adults. 

## Materials and methods

Study design and data source

This study was a cross-sectional secondary analysis of data from the 2005-2006 NHANES. NHANES is conducted by the National Center for Health Statistics, Hyattsville, Maryland, United States, and uses a complex, multistage, stratified probability sampling design to collect demographic, questionnaire, examination, and laboratory information from the United States civilian non-institutionalized population [12,13]. Data from the NHANES 2005-2006 Demographics, Physical Activity Questionnaire, Diabetes Questionnaire, and Body Measures files were merged using the unique participant identifier SEQN. This study used publicly available, de-identified NHANES data and therefore did not require additional institutional review board approval or additional informed consent.

Study population

The 2005-2006 NHANES cycle included 10,348 participants. The present analysis was restricted to adults aged 18 years or older with available information on PA, diabetes status, demographic and socioeconomic characteristics, and measured body mass index (BMI). Participants younger than 18 years, pregnant participants, and participants with missing or incomplete information required for the analysis were excluded. The final complete-case analytic sample consisted of 8,174 adults. Because individual exclusion counts for each criterion were not retained in the available analytic records, this is acknowledged as a reporting limitation.

Physical activity assessment

PA was assessed using self-reported information from the NHANES 2005-2006 Physical Activity Questionnaire. The NHANES PA component collected information regarding transportation-related activity, daily activity, and moderate and vigorous leisure-time PA.

Participants were categorized according to their calculated amount of moderate-to-vigorous PA as low, moderate, or high PA. Low PA was defined as less than 150 minutes per week, moderate PA as 150-300 minutes per week, and high PA as more than 300 minutes per week. Vigorous-intensity activity was given twice the weight of moderate-intensity activity when calculating the combined activity measure.

The 2005-2006 NHANES PA files include variables describing transportation activity and moderate and vigorous activity. The detailed individual-activity file additionally contains the reported activity, intensity level, frequency during the preceding 30 days, duration per activity session, and metabolic equivalent value. NHANES excluded reported leisure-time activities lasting less than 10 minutes and treated implausible activity durations according to its predefined data-processing procedures.

Diabetes outcome

Diabetes status was determined using the NHANES Diabetes Questionnaire. The primary self-report item, DIQ010, asks participants whether a doctor or other health professional had ever told them that they had diabetes or sugar diabetes, excluding diabetes occurring only during pregnancy. Participants answering yes were classified as having self-reported diabetes, and those answering no were classified as not having self-reported diabetes. Responses indicating borderline diabetes were not classified as diabetes in the primary analysis.

Because the self-reported diabetes questionnaire item does not by itself reliably distinguish type 2 diabetes from type 1 or other forms of diabetes, the outcome is described as self-reported diabetes or prevalent self-reported diabetes rather than confirmed type 2 diabetes. No claim of laboratory validation is made in the revised analysis.

Covariates

Demographic covariates included age, sex, and race or ethnicity. Socioeconomic covariates included education level and annual household income. Household income was categorized as low income, less than 20,000 dollars annually; middle income, 20,000 to 44,999 dollars annually; and high income, 45,000 dollars or more annually. 

BMI was calculated from measured height and weight and expressed in kilograms per square meter. BMI categories were defined as underweight (less than 18.5 kg/m^2^); normal weight (18.5-24.9 kg/m^2^); overweight (25.0-29.9 kg/m^2^); and obesity (30.0 kg/m^2^ or greater). BMI was included as an additional covariate in the final sequential regression model. A formal mediation analysis was not performed; therefore, BMI adjustment is interpreted only as additional statistical adjustment and not as evidence of mediation or an indirect causal pathway.

Statistical analysis

Statistical analyses were performed using IBM SPSS Statistics for Windows, version 26.0 (IBM Corp., Armonk, New York, United States). Continuous variables were summarized using mean and standard deviation (SD), and categorical variables were summarized using frequencies and percentages. Differences across PA categories were assessed using one-way analysis of variance for continuous variables and chi-square tests for categorical variables.

Binary logistic regression was used to examine the association between PA category and prevalent self-reported diabetes, with low PA serving as the reference category. Four sequential models were evaluated. Model 1 was unadjusted. Model 2 was adjusted for age, sex, and race or ethnicity. Model 3 was additionally adjusted for education and household income. Model 4 additionally included BMI.

Odds ratios (ORs) with 95% confidence intervals (CIs) were reported. All statistical tests were two-sided, and a p-value of less than 0.05 was considered statistically significant.

The analyses presented in this study were not documented as incorporating NHANES examination weights, masked variance strata, or primary sampling units. Accordingly, the findings are interpreted as unweighted associations within the analyzed NHANES 2005-2006 sample rather than as nationally representative population estimates. The lack of documented complex-survey adjustment is acknowledged as a limitation. Subgroup analyses, formal interaction testing, sensitivity analyses, and formal mediation analyses are not reported because these analyses were not documented in the available results.

## Results

The final analytic sample included 8,174 adults aged 18 years or older from the NHANES 2005-2006 cycle. The mean age was 44.9 ± 17.9 years, and the mean BMI was 28.2 ± 6.2 kg/m^2^. A total of 4,349 participants (53.2%) were male, while 3,825 participants (46.8%) were female. Among the participants, 54.2% were non-Hispanic White, 23.3% were non-Hispanic Black, 15.4% were Mexican American, 2.9% were other Hispanic, and 4.3% were classified as other race or multiracial. Regarding education, 7.2% had less than a ninth-grade education, 11.3% had completed grades 9 through 11, 20.8% had completed high school or a GED, 33.1% had some college education or an associate degree, and 27.5% were college graduates or above. Household income was categorized as low for 18.8% of participants, middle for 41.3%, and high for 39.9%. With respect to PA, 3,019 participants (36.9%) were classified as having low PA, 1,236 participants (15.1%) as having moderate PA, and 3,919 participants (47.9%) as having high PA. Overall, 650 participants (8.0%) were classified as having self-reported diabetes, while 7,524 participants (92.0%) were classified as not having diabetes. The characteristics of the overall analytic sample are presented in Table 1.

**Table 1:** Descriptive statistics of the overall analytic sample (N=8,174) Categorical variables are presented as frequencies and percentages. Continuous variables are presented as mean ± standard deviation.

Characteristic	Value
Age (years), mean ± SD	44.9±17.9
BMI (kg/m²), mean ± SD	28.2 ± 6.2
Gender, n (%)	
Male	4,349 (53.2%)
Female	3,825 (46.8%)
Race/Ethnicity, n (%)	
Non-Hispanic White	4,430 (54.2%)
Non-Hispanic Black	1,905 (23.3%)
Mexican American	1,259 (15.4%)
Other Hispanic	237 (2.9%)
Other Race/Multi-Racial	352 (4.3%)
Education Level, n (%)	
<9th grade	589 (7.2%)
9-11th grade	924 (11.3%)
High School/GED	1,700 (20.8%)
Some College/Associate	2,706 (33.1%)
College Graduate or Above	2,248 (27.5%)
Income Category, n (%)	
Low Income	1,537 (18.8%)
Middle Income	3,376 (41.3%)
High Income	3,261 (39.9%)
Physical Activity Level, n (%)	
Low	3,019 (36.9%)
Moderate	1,236 (15.1%)
High	3,919 (47.9%)
Diabetes Status, n (%)	
No Diabetes	7,524 (92.0%)
Diabetes	650 (8.0%)

Participant characteristics differed across physical activity categories. The mean age was 50.3 ± 18.6 years in the low PA group, 44.5 ± 17.7 years in the moderate PA group, and 40.8 ± 16.2 years in the high PA group. This difference was statistically significant with an F value of 256.0 and a p value of less than 0.001. The distribution of sex also differed significantly across PA categories. Male participants represented 46.0% of the low activity group, 51.4% of the moderate activity group, and 59.3% of the high activity group. The corresponding percentages for female participants were 54.0%, 48.6%, and 40.7%, respectively. The chi-square value was 123.4 with a p-value of less than 0.001. Race or ethnicity, education level, and household income also differed significantly across physical activity categories, with chi-square values of 68.8, 251.1, and 138.9, respectively, and p-values of less than 0.001. Mean BMI was 28.9 ± 6.7 kg/m^2^ among participants with low PA, 27.6 ± 5.7 kg/m^2^ among those with moderate PA, and 27.9 ± 5.9 kg/m^2^ among those with high PA. This difference was statistically significant with an F value of 29.8 and a p-value of less than 0.001.

Self-reported diabetes was present in 356 of 3,019 participants (11.8%) in the low PA group, compared with 73 of 1,236 participants (5.9%) in the moderate PA group and 221 of 3,919 participants (5.7%) in the high PA group. The distribution of diabetes status differed significantly across PA categories with a chi-square value of 96.5 and a p-value of less than 0.001. Because the prevalence estimates in the moderate and high PA groups were similar, these results should not be interpreted as demonstrating a monotonic dose-response relationship. Detailed comparisons are presented in Table 2. The association between PA and diabetes status showed a significant inverse relationship (p<0.001), as among subjects with low PA, 356 (11.8%) had diabetes, compared to 73 (5.9%) in the moderate PA group, and 221 (5.7%) in the high PA group.

**Table 2:** Participant characteristics stratified by physical activity level Categorical variables are presented as frequencies and percentages and were compared using chi-square tests. Continuous variables are presented as mean ± SD and were compared using one-way analysis of variance *p value of less than 0.05 was considered statistically significant

Characteristic	Physical activity			Test value	p-value
	Low	Moderate	High		
Number of participants, n (%)	3,019 (36.9%)	1,236 (15.1%)	3,919 (47.9%)	-	-
Age (years), mean±SD	50.3 ± 18.6	44.5 ± 17.7	40.8 ± 16.2	F=256.0	<0.001*
Sex, n (%)				X^2^=123.4	<0.001*
Male	1,389 (46.0%)	635 (51.4%)	2,325 (59.3%)		
Female	1,630 (54.0%)	601 (48.6%)	1,594 (40.7%)		
Race/Ethnicity, n (%)				X^2^=68.8	<0.001*
Non-Hispanic White	1,504 (49.8%)	697 (56.4%)	2,229 (56.9%)		
Non-Hispanic Black	824 (27.3%)	271 (21.9%)	810 (20.7%)		
Mexican American	503 (16.7%)	177 (14.3%)	579 (14.8%)		
Other Hispanic	93 (3.1%)	35 (2.8%)	109 (2.8%)		
Other Race/Multi-Racial	95 (3.1%)	56 (4.5%)	192 (4.9%)		
Education Level, n (%)				X^2^=251.1	<0.001*
Less than 9th grade	325 (10.8%)	73 (5.9%)	191 (4.9%)		
9-11th grade	438 (14.5%)	126 (10.2%)	360 (9.2%)		
High School/GED	698 (23.1%)	246 (19.9%)	756 (19.3%)		
Some College/Associate	957 (31.7%)	424 (34.3%)	1,325 (33.8%)		
College Graduate or Above	601 (19.9%)	367 (29.7%)	1,287 (32.8%)		
Income Category, n (%)				X^2^=138.9	<0.001*
Low Income	728 (24.1%)	203 (16.4%)	606 (15.5%)		
Middle Income	1,304 (43.2%)	498 (40.3%)	1,574 (40.2%)		
High Income	987 (32.7%)	535 (43.3%)	1,739 (44.4%)		
BMI (kg/m²)	28.9 ± 6.7	27.6 ± 5.7	27.9 ± 5.9	F=29.8	<0.001^
Diabetes Status, n (%)				X^2^=96.5	<0.001*
No Diabetes	2,663 (88.2%)	1,163 (94.1%)	3,698 (94.3%)		
Diabetes	356 (11.8%)	73 (5.9%)	221 (5.7%)		

In the unadjusted logistic regression model, moderate PA was associated with 53% lower odds of diabetes (OR 0.47, 95%CI 0.36-0.61, p<0.001) and high PA with 55% lower odds (OR 0.45, 95%CI 0.38-0.54, p-value <0.001), compared with low PA. 

After adjustment for age, sex, and race or ethnicity in Model 2, the associations were attenuated but remained statistically significant, as moderate PA was associated with 36% lower odds (OR 0.64, 95%CI 0.49-0.84, p-value 0.001), and high PA with 30% lower odds (OR 0.70, 95%CI 0.58-0.85, p-value <0.001).

After additional adjustment for education and household income in Model 3, the associations persisted as moderate PA was associated with 33% lower odds of diabetes (OR 0.67, 95%CI 0.51-0.89, p-value 0.005), and high PA with 26% lower odds (OR 0.74, 95%CI 0.61-0.90, p-value 0.002). Low-income (OR 1.33, 95%CI 1.03-1.72, p-value 0.026) and middle-income (OR 1.28, 95%CI 1.04-1.59, p-value 0.022) subjects had significantly higher odds of diabetes compared with high-income participants. 

In Model 4 (fully adjusted, including BMI), PA remained protective though attenuated, as moderate PA was associated with 25% lower odds (OR 0.75, 95%CI 0.57-0.99, p-value 0.042), and high PA with 19% lower odds (OR 0.81, 95%CI 0.66-0.99, p-value 0.048). BMI emerged as a strong independent predictor, with each one-unit increase in BMI associated with 9% higher odds of diabetes (OR 1.09, 95%CI 1.07-1.10, p-value <0.001). Age remained significant, with each additional year associated with 5% higher odds (OR 1.05, 95%CI 1.05-1.06, p-value <0.001) of diabetes. The attenuation observed after adjustment for BMI is interpreted as additional statistical adjustment and not as evidence of mediation or a causal pathway. Logistic regression results are summarized in Table 3.

**Table 3:** Logistic regression models for the association between physical activity level and self-reported diabetes Low physical activity served as the reference category. Model 1 was unadjusted. Model 2 was adjusted for age, sex, and race or ethnicity. Model 3 was additionally adjusted for education and household income. Model 4 was additionally adjusted for BMI.

Physical Activity Level	Model 1, OR (95% CI)	Model 2, OR (95% CI)	Model 3, OR (95% CI)	Model 4, OR (95% CI)
Low (Reference)	1.00	1.00	1.00	1.00
Moderate	0.47 (0.36-0.61)	0.64 (0.49-0.84)	0.67 (0.51-0.89)	0.75 (0.57-0.99)
High	0.45 (0.38-0.54)	0.70 (0.58-0.85)	0.74 (0.61-0.90)	0.81 (0.66-0.99)

## Discussion

In this cross-sectional secondary analysis of NHANES 2005-2006 data, both moderate and high levels of self-reported PA were associated with lower odds of prevalent self-reported diabetes compared with low PA. In the fully adjusted model, moderate PA was associated with 25% lower odds of diabetes, while high PA was associated with 19% lower odds. The magnitude of these associations was attenuated after adjustment for demographic and socioeconomic characteristics and BMI. Because PA and diabetes status were assessed within the same survey cycle, these findings demonstrate an association and do not establish temporality or causality.

The attenuation of the association after adjustment for BMI suggests that BMI is related to the observed relationship between PA and diabetes; however, the present analysis cannot determine whether BMI acts as a mediator or represents a specific biological pathway. PA and adiposity may be related to diabetes through complex and potentially bidirectional mechanisms. Previous studies have demonstrated that aerobic and resistance exercise can improve insulin sensitivity and glucose metabolism [14-16], but the current cross-sectional findings should not be interpreted as evidence of a direct physiological effect.

The direction of the observed association is generally consistent with previous research examining PA and diabetes. A meta-analysis involving more than 300,000 participants reported a lower risk of type 2 diabetes among individuals engaging in moderate PA compared with those who were inactive [17]. Smith and colleagues also reported a nonlinear association between PA and incident type 2 diabetes in prospective cohort studies [18]. In the present study, however, diabetes prevalence was similar in the moderate and high PA groups, at 5.9% and 5.7%, respectively, and the fully adjusted OR was slightly closer to the null for high activity than for moderate activity. Therefore, the present findings should not be interpreted as demonstrating a monotonic dose-response relationship.

Other population-based studies have similarly reported inverse associations between PA and diabetes. Dehdari et al. reported differences in diabetes prevalence according to PA level in an Iranian adult population [19]. Ghaderpanahi et al. found that moderate aerobic PA was associated with lower odds of diabetes, although the magnitude of association differed according to obesity status [20]. In the present analysis, adjustment for BMI also attenuated the association between PA and diabetes, although the cross-sectional design prevents conclusions about the direction or mechanism of this relationship.

Prospective evidence also provides useful context. Yang et al. reported that moderate and high PA were associated with a lower incidence of diabetes among adults with prediabetes during follow-up [21]. These prospective findings cannot be directly compared with the present cross-sectional prevalence analysis because the study designs and populations differ. Similarly, findings from the Diabetes Prevention Program demonstrated a substantial reduction in diabetes incidence among high-risk individuals receiving an intensive lifestyle intervention that included PA, dietary modification, and weight-loss goals [11]. Unlike that randomized intervention, the present study evaluated self-reported habitual PA and prevalent diabetes at a single time point and therefore cannot determine whether increasing PA reduces future diabetes incidence.

PA may also be relevant among individuals who already have diabetes. Bukht et al. reported a higher prevalence of diabetes-related complications among physically inactive patients compared with active patients [22]. In addition, Xiao et al., using accelerometer-based data from NHANES 2011-2014, reported that the timing of PA was associated with diabetes status [23]. Together, these studies demonstrate that the relationship between PA and diabetes has been examined across different populations, measurement approaches, and study designs.

Older age was associated with higher odds of self-reported diabetes in the present analysis. This finding is consistent with previous evidence demonstrating a greater burden of diabetes among older adults [24]. Age-related changes in glucose metabolism, beta-cell function, and insulin sensitivity may contribute to this association [25]. Previous research also indicates that PA remains metabolically beneficial among older adults [26]. Nevertheless, because age and PA were measured concurrently in this analysis, the present findings cannot determine the temporal relationship among age-related changes, activity level, and diabetes.

In the present study, participants in the low- and middle-income categories had higher odds of self-reported diabetes compared with those in the high-income category. Previous research has linked socioeconomic disadvantage with differences in opportunities for PA, dietary patterns, chronic stress, healthcare access, and other social determinants of health [27,28]. The observed income differences in this analysis may therefore reflect multiple social, behavioral, and clinical factors that were not completely measured or controlled.

Limitations and strengths

Several limitations should be considered when interpreting these findings. First, the cross-sectional design prevents determination of temporal sequence and does not allow causal inference. Reverse causation is possible because individuals with diabetes, diabetes-related symptoms, comorbidities, or treatment-related limitations may reduce their PA after diagnosis. Second, PA was self-reported and is therefore susceptible to recall error and misclassification. Diabetes status was also based primarily on self-report, and this measure cannot reliably distinguish type 2 diabetes from type 1 or other forms of diabetes; therefore, we refer to the outcome as self-reported diabetes rather than confirmed type 2 diabetes.

Third, the analysis used a complete-case approach, and exclusion of participants with missing information may have introduced selection bias. Individual counts for each exclusion criterion were not retained in the available analytic records, limiting detailed evaluation of the participant-selection process. Fourth, residual confounding remains possible because potentially important factors, including dietary intake, family history of diabetes, medication use, comorbidities, and other lifestyle characteristics, were not fully incorporated into the analysis.

Fifth, the analyses reported in this study were not documented as incorporating NHANES sampling weights, masked variance strata, or primary sampling units. Consequently, the estimates should be interpreted as associations within the analyzed NHANES 2005-2006 sample rather than as nationally representative population estimates. Failure to account for the complex survey design may also affect variance estimates and statistical precision. Finally, the 2005-2006 NHANES cycle represents an older population sample, and patterns of PA, diabetes prevalence, treatment, and other population characteristics may have changed over time.

Despite these limitations, the study evaluates a clinically and public-health relevant association in a large United States dataset and presents sequentially adjusted models incorporating several demographic and socioeconomic characteristics. The findings demonstrate that moderate and high levels of self-reported PA were associated with lower odds of prevalent self-reported diabetes in the analyzed sample. Prospective studies using objective measures of PA and appropriate complex-survey methods are needed to evaluate temporal relationships and determine whether changes in PA are associated with subsequent diabetes incidence.

## Conclusions

In this cross-sectional secondary analysis of the NHANES 2005-2006 sample, moderate and high levels of self-reported PA were associated with lower odds of prevalent self-reported diabetes compared with low PA. These associations remained after adjustment for demographic, socioeconomic, and BMI-related factors, although the estimates were attenuated after BMI adjustment.

Because the study was cross-sectional, temporality and causality cannot be established, and the findings should not be interpreted as evidence that increasing PA directly reduces diabetes incidence or burden. Prospective studies using appropriate complex-survey methods and objective measures of PA are needed to evaluate whether changes in PA are associated with subsequent diabetes risk.

## References

[1] (2023). Global, regional, and national burden of diabetes from 1990 to 2021, with projections of prevalence to 2050: a systematic analysis for the Global Burden of Disease Study 2021. Lancet.

[2] Magliano DJ, Boyko EJ (2021). IDF Diabetes Atlas. International Diabetes Federation, Brussels.

[3] (2024). Centers for Disease Control and Prevention: National Diabetes Statistics Report. https://www.cdc.gov/diabetes/php/data-research/index.html.

[4] Parker ED, Lin J, Mahoney T (2024). Economic costs of diabetes in the U.S in 2022. Diabetes Care.

[5] Galaviz KI, Narayan KM, Lobelo F, Weber MB (2018). Lifestyle and the prevention of type 2 diabetes: a status report. Am J Lifestyle Med.

[6] Hamasaki H (2016). Daily physical activity and type 2 diabetes: a review. World J Diabetes.

[7] Kanaley JA, Colberg SR, Corcoran MH (2022). Exercise/physical activity in individuals with type 2 diabetes: a consensus statement from the American College of Sports Medicine. Med Sci Sports Exerc.

[8] Bird SR, Hawley JA (2016). Update on the effects of physical activity on insulin sensitivity in humans. BMJ Open Sport Exerc Med.

[9] Liu Z, Guo Y, Zheng C (2024). Type 2 diabetes mellitus related sarcopenia: a type of muscle loss distinct from sarcopenia and disuse muscle atrophy. Front Endocrinol (Lausanne).

[10] LeBrasseur NK, Walsh K, Arany Z (2011). Metabolic benefits of resistance training and fast glycolytic skeletal muscle. Am J Physiol Endocrinol Metab.

[11] (2002). The Diabetes Prevention Program (DPP): description of lifestyle intervention. Diabetes Care.

[12] (2026). National Health and Nutrition Examination Survey 2005-2006 Demographics Data Documentation, Codebook, and Frequencies. Demographic Variables & Sample Weights (DEMO_D). https://wwwn.cdc.gov/Nchs/Data/Nhanes/Public/2005/DataFiles/DEMO_D.htm.

[13] (2026). National Health and Nutrition Examination Survey 2005-2006 Demographics Data Documentation, Codebook, and Frequencies. Physical Activity (PAQ_D). https://wwwn.cdc.gov/Nchs/Data/Nhanes/Public/2005/DataFiles/PAQ_D.htm.

[14] Merz KE, Thurmond DC (2020). Role of skeletal muscle in insulin resistance and glucose uptake. Compr Physiol.

[15] Richter EA (2021). Is GLUT4 translocation the answer to exercise-stimulated muscle glucose uptake?. Am J Physiol Endocrinol Metab.

[16] Denche-Zamorano Á, Mendoza-Muñoz DM, Barrios-Fernandez S (2022). Physical activity reduces the risk of developing diabetes and diabetes medication use. Healthcare (Basel).

[17] Jeon CY, Lokken RP, Hu FB, van Dam RM (2007). Physical activity of moderate intensity and risk of type 2 diabetes: a systematic review. Diabetes Care.

[18] Smith AD, Crippa A, Woodcock J, Brage S (2016). Physical activity and incident type 2 diabetes mellitus: a systematic review and dose-response meta-analysis of prospective cohort studies. Diabetologia.

[19] Dehdari T, Shahraki-Sanavi F, Nasiri A, Nouri R, Moghaddam AA, Mohammadi M (2025). Relationship between physical activity levels and type 2 diabetes in adults: Zahedan Adult Cohort Study, Iran. Health Sci Rep.

[20] Ghaderpanahi M, Fakhrzadeh H, Sharifi F (2011). Association of physical activity with risk of type 2 diabetes. Iran J Public Health.

[21] Yang W, Wu Y, Chen Y, Chen S, Gao X, Wu S, Sun L (2024). Different levels of physical activity and risk of developing type 2 diabetes among adults with prediabetes: a population-based cohort study. Nutr J.

[22] Bukht MS, Ahmed KR, Hossain S, Masud P, Sultana S, Khanam R (2019). Association between physical activity and diabetic complications among Bangladeshi type 2 diabetic patients. Diabetes Metab Syndr.

[23] Xiao Q, Feng Q, Rutter MK, Albalak G, Wang H, Noordam R (2025). Associations between the timing of 24 h physical activity and diabetes mellitus: results from a nationally representative sample of the US population. Diabetologia.

[24] Kirkman MS, Briscoe VJ, Clark N (2012). Diabetes in older adults. Diabetes Care.

[25] Chia CW, Egan JM, Ferrucci L (2018). Age-related changes in glucose metabolism, hyperglycemia, and cardiovascular risk. Circ Res.

[26] Ryan AS (2010). Exercise in aging: its important role in mortality, obesity and insulin resistance. Aging health.

[27] Walker RJ, Smalls BL, Campbell JA, Strom Williams JL, Egede LE (2014). Impact of social determinants of health on outcomes for type 2 diabetes: a systematic review. Endocrine.

[28] Hosseini Shokouh SM, Arab M, Emamgholipour S, Rashidian A, Montazeri A, Zaboli R (2017). Conceptual models of social determinants of health: a narrative review. Iran J Public Health.

